# Febuxostat‐Induced Agranulocytosis: An Illustrative Case Report and Comprehensive Literature Review

**DOI:** 10.1002/ccr3.70846

**Published:** 2025-09-10

**Authors:** Qiuping Liu, Bing Zhong

**Affiliations:** ^1^ Department of Rheumatology and Immunology First Affiliated Hospital of Army Medical University Chongqing China

**Keywords:** adverse reaction, febuxostat, granulocytosis, hyperuricemia

## Abstract

Neutropenia induced by febuxostat is uncommon, with limited literature available to assess its frequency. This rare complication carries a serious risk to life. It is essential for healthcare providers to stay alert and informed about this potential adverse reaction.

## 1 Introduction

Lowering uric acid levels is widely accepted as the main treatment for hyperuricemia. The American College of Rheumatology guidelines suggest xanthine oxidase inhibitors, such as febuxostat, as the initial treatment option for hyperuricemia and are frequently prescribed [1]. Febuxostat differs from the traditional xanthine oxidase inhibitor allopurinol in that it reduces uric acid production through a non‐competitive mechanism. This medication is primarily broken down in the liver through the creation and oxidation of glucuronide salts. These characteristics provide substantial benefits in lowering uric acid levels without interfering with other enzymes related to purine or pyrimidine metabolism at recommended doses [2, 3]. Hence, febuxostat presents a compelling alternative, particularly for individuals who are unable to tolerate allopurinol. Common side effects of febuxostat include abnormal liver function, nausea, and rash. With the increasing use of febuxostat in clinical practice, rare adverse reactions like agranulocytosis may become more apparent. Agranulocytosis is a serious condition where the neutrophil count drops below 0.5 × 10^9^/L and can be life‐threatening. This report highlights a case of severe agranulocytosis linked to febuxostat treatment and provides a thorough overview of febuxostat‐induced agranulocytosis reported in medical literature, with the goal of promoting the appropriate use of this drug in clinical settings.

## 2 Case History/Examination

A 20‐year‐old male with ankylosing spondylitis and hyperuricemia was admitted for further investigation and treatment due to agranulocytosis. The patient is currently showing vital signs as follows: temperature of 36.2°C, pulse rate of 91 beats per minute, respiratory rate of 20 breaths per minute, blood pressure of 132/75 mmHg, height measuring 172.5 cm, and weight recorded as 97.1 kg. He is experiencing normal growth, has a well‐balanced diet, and is at a healthy weight proportional to his height. Prior to this, the patient was regularly taking thalidomide 50 mg once daily and celecoxib 0.2 g twice daily, with the ankylosing spondylitis being reasonably controlled. Upon finding a serum uric acid concentration of 532 μmol/L, treatment with febuxostat 40 mg daily was initiated. Except for febuxostat, all other medications had been taken for over 6 months. Prior to commencing febuxostat therapy, the patient had a white blood cell count of 5.21 × 10^9^/L and a neutrophil count of 2.19 × 10^9^/L in their laboratory results. Following 4 weeks of febuxostat treatment, the patient's blood tests indicated a white blood cell count of 3.29 × 10^9^/L and a neutrophil count of 0.34 × 10^9^/L. Additionally, the patient exhibited normal vital signs, including pulse and temperature, and no significant abnormalities were detected during cardiovascular, pulmonary, and abdominal examinations. The patient denied experiencing any other specific discomforts. He reported no history of acute leukemia, multiple myeloma, or other blood disorders, and confirmed that he was not taking any medications related to these conditions. Furthermore, tests for EB virus and HIV infection came back negative, and screenings for autoimmune‐related antibodies such as antinuclear antibodies (ANA) and a panel of 15 autoantibodies were also negative.

## 3 Methods

A bone marrow biopsy revealed a lower proportion of neutrophils but normal bone marrow cells, indicating granulocytopenia without any signs of blood cancer. In order to eliminate the possibility of primary hematologic disorders, a consultation was requested with a hematologist. They proposed that the granulocytopenia could be a result of drug‐induced bone marrow suppression. Due to the potential link between agranulocytosis and febuxostat, the medication was promptly halted. The patient's treatment regimen was adjusted to include Qi‐Ji‐Sheng‐Bai capsules at a dosage of 2 g three times daily, along with Leucogen 20 mg three times a day. Additionally, a 3‐day course of G‐CSF therapy was administered. Given the likelihood of agranulocytosis stemming from immune or cytotoxic mechanisms, which could heighten susceptibility to infections, infection prevention measures were underscored throughout the treatment. Following a 4‐day hospital stay, the patient's peripheral neutrophil count notably improved, with neutrophils rising from 0.34 × 10^9^/L to 2.85 × 10^9^/L, and the white blood cell count increasing to 7.54 × 10^9^/L. The patient's blood work returned to normal, ankylosing spondylitis was effectively managed, G‐CSF treatment was discontinued, and the patient was discharged for routine outpatient monitoring.

**FIGURE 1:**
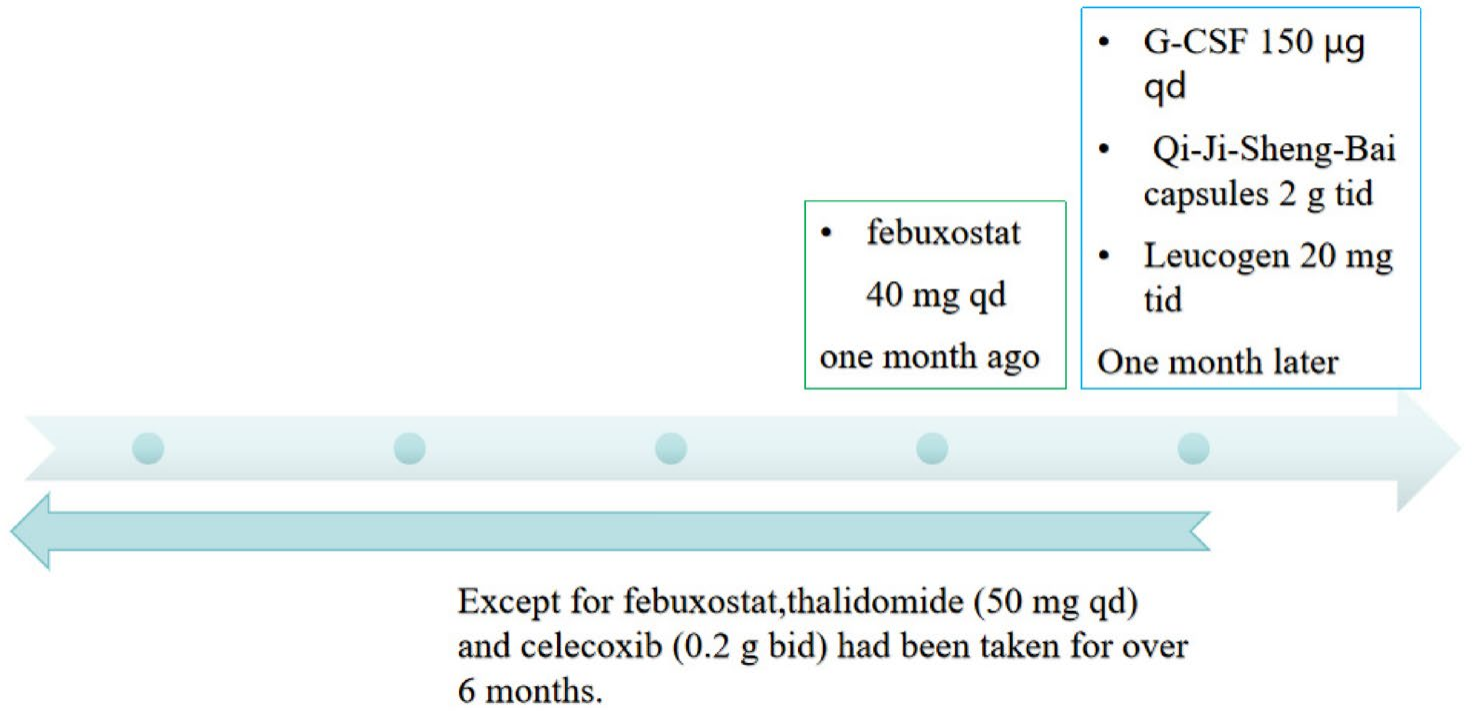
The medication history before and after the onset of agranulocytosis.

**FIGURE 2:**
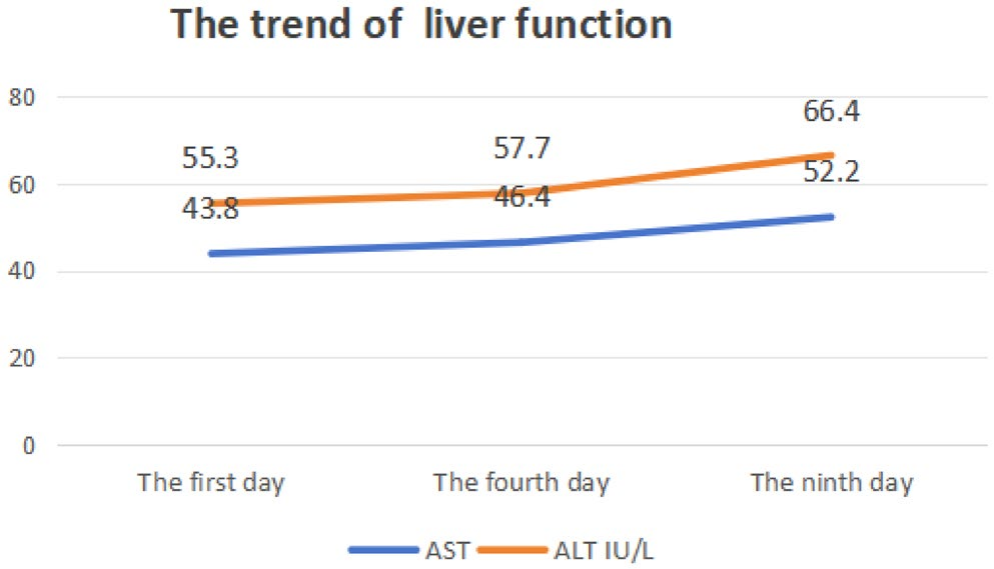
The trend of liver function.

**FIGURE 3:**
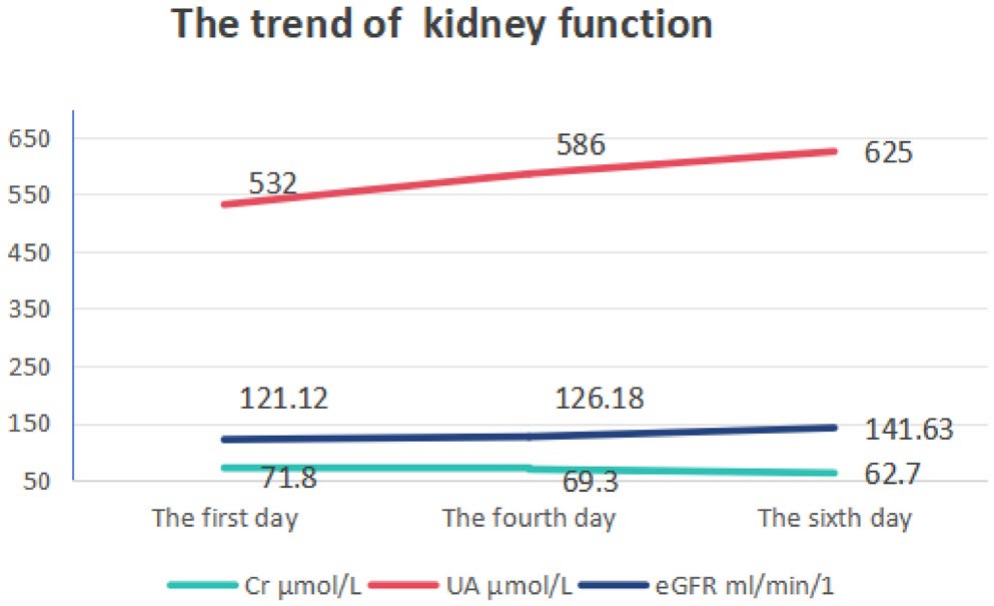
The trend of kidney function.

**FIGURE 4:**
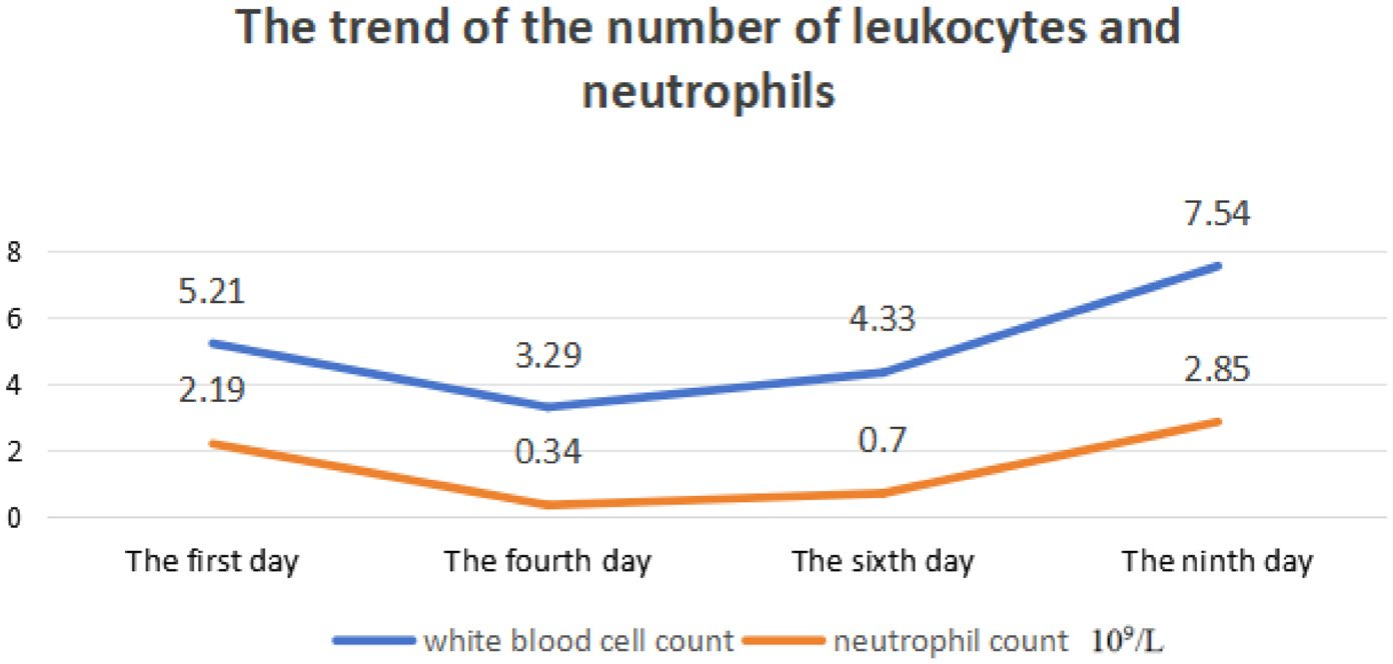
The trend of the number of leukocytes and neutrophils.

The Naranjo probability scale was used to evaluate the possibility of a causal connection between agranulocytosis and febuxostat in patients (Table 1). Figure 1 shows the medication status of the patient before and after neutropenia; Figure 2 shows the changes in the patient's liver function; Figure 3 shows the changes in the patient's kidney function; and Figure 4 shows the changes in the patient's white blood cells and neutrophils.

**TABLE 1:** Naranjo score.

Question	Yes	No	Unknown	Score
1. Have there been any definitive reports on this reaction in the past?	+1	0	0	+1
2. Did the adverse event improve after discontinuing the drug or administering a specific antagonist?	+2	−1	0	+2
3. Was there an improvement in the adverse event after stopping the drug or giving a specific antagonist?	+1	0	0	+1
4. Was the adverse event observed again when the drug was administered a second time?	+2	−1	0	0
5. Are there other possible reasons that could have independently triggered the reaction?	−1	+2	0	0
6. Was there a recurrence of the reaction when a placebo was administered?	−1	+1	0	0
7. Was the drug found in blood or other fluids at levels that are known to be harmful?	+1	0	0	0
8. Did the reaction worsen with a higher dose or improve with a lower dose?	+1	0	0	0
9. Has the patient experienced a comparable reaction to the same or similar medications in any previous instances?	+1	0	0	0
10. Was there any objective evidence confirming the adverse event?	+1	0	0	+1
Total score				5

*Note:* According to the Naranjo score results, total score ≥ 9, definite; 5–8, highly probable; 1–4, possible; ≤ 0, suspicious.

## 4 Discussion

Febuxostat has been approved for the treatment of hyperuricemia in gout patients in the United States, for chronic hyperuricemia with uric acid deposition in the European Union, and for hyperuricemia in patients with or without gout in Japan [4]. With the increasing use of febuxostat in populations with hyperuricemia, it has been shown to lower serum uric acid levels in clinical settings. However, it is important to note that there are potential toxic risks associated with febuxostat, specifically immune‐related adverse events. Abnormal liver function, nausea, and rash are the most common adverse reactions associated with the use of febuxostat. Less reported adverse events include agranulocytosis, which is difficult to assess for its overall incidence due to the small number of cases. A literature review of the PubMed database revealed only three cases of neutropenia potentially attributed to febuxostat [5, 6]: A 67‐year‐old woman with gout and end‐stage renal disease (ESRD) was given a prescription for 40 mg of febuxostat daily for 2.5 months. Subsequently, she experienced febrile neutropenia, leading to a significant decrease in her absolute neutrophil count to only 0.03 × 10^9^/L; during hospitalization, a 74‐year‐old woman with cirrhosis and chronic kidney disease (CKD) received febuxostat treatment for hyperuricemia. After 11 days of febuxostat therapy, the patient experienced neutropenia, as indicated by a blood test revealing a neutrophil count of 0.6 × 10^9^/L; a 68‐year‐old man with type 2 diabetes and intermittent hemodialysis was admitted to the hospital and prescribed febuxostat for hyperuricemia. After 3 days of using febuxostat, he developed neutropenia, indicated by a decrease in neutrophil count from 5.7 × 10^9^/L to 0.5 × 10^9^/L on laboratory tests. The case report highlights the importance of promptly stopping any medications that could lead to severe hematologic toxicity and replacing them with safer alternatives. Patients should be closely monitored in a hospital setting with infection control measures or undergo rigorous clinical and microbiological monitoring in the hematology department to prevent further complications. The recommended course of action involves the use of G‐CSF for treatment. In cases of fever or sepsis, immediate administration of antibiotics in accordance with treatment protocols is advised. Prompt initiation of appropriate pharmacological treatment, whether individual or combined, can significantly reduce recovery time and hospital stay duration. It is crucial for healthcare providers to be vigilant about the rare yet potentially life‐threatening adverse reaction associated with febuxostat, given its widespread clinical use. While most patients recover within a few days of treatment, clinicians should carefully evaluate the decision to continue suspicious drug therapy in cases where data is lacking, balancing the potential benefits and risks. Patients may encounter abnormal blood cell counts again, and the continuation or rechallenge of treatment could escalate these risks.

This article details the fourth confirmed instance of agranulocytosis linked to the usage of febuxostat. The individual, diagnosed with ankylosing spondylitis, developed agranulocytosis after undergoing febuxostat treatment for hyperuricemia. Upon suspecting a connection between febuxostat and agranulocytosis, the medication was discontinued, and the patient was treated symptomatically with G‐CSF. Subsequently, the neutrophil count normalized. The patient had no prior history of blood disorders, and a bone marrow biopsy ruled out other potential causes of pancytopenia. To establish the association between febuxostat and agranulocytosis, we utilized the Naranjo scoring system for analysis (Table 1). With a combined score of 5 points, there is a strong indication of a relationship between the two factors. Upon reviewing the drug manual and the patient's medication history, it was found that the other medications being taken had been used for a prolonged period with no clear connection to the onset of this adverse reaction. Based on the timing and recurring nature of agranulocytosis episodes, it is plausible that febuxostat may be the cause of agranulocytosis in this individual.

Neutrophils are a significant component of white blood cells, accounting for approximately 50%–70% of peripheral white blood cells in the human body. They primarily mediate the inflammatory process through phagocytosis, intracellular degradation, granule release, and the formation of neutrophil extracellular traps to destroy invading pathogens, serving as an important defense line of the human immune system [7]. Agranulocytosis is a serious and potentially life‐threatening form of neutrophil deficiency, characterized by a neutrophil count below 0.5 × 10^9^/L. It is not only a risk factor for infectious complications but also a manifestation of immune dysfunction. Usually, agranulocytosis is asymptomatic before the onset of infection. Fatigue, fever, chills, muscle and joint pain are typically the initial symptoms of the disease. These symptoms are often initially overlooked because they are commonly mistaken for flu‐like infections. Agranulocytosis commonly presents with symptoms including fever, a sore throat, difficulty swallowing, and mucosal lesions like ulcers [8]. Literature reports that about 60% of agranulocytosis in clinical practice is caused by drugs [9]. Typically, agranulocytosis manifests within 1 week to 10 days after the suspected drug is initiated, primarily due to allergic reactions. In instances of agranulocytosis caused by other mechanisms, it can take several weeks for the toxic effects on bone marrow hematopoietic stem cells to manifest. Overall, the exact mechanism behind drug‐induced agranulocytosis remains unclear. According to this article and three cases in the literature, as well as the evaluation on the Naranjo scale, we believe that agranulocytosis can be attributed to several factors. These include myelosuppression, where drugs' active metabolites may harm bone marrow cells, immune‐mediated reactions triggered by drug‐protein complexes, genetic susceptibility impacting drug metabolism, and factors such as dosage, treatment duration, and overall health status. A high percentage of the four patients are elderly, whose declining organ and system functions can impact how drugs are processed in the body, potentially increasing the risk of adverse reactions [10]. Taken together, these interactions may play a role in the development of agranulocytosis.

## 5 Conclusion

The literature and case study presented here show that agranulocytosis can develop in individuals of all ages when taking febuxostat. The period of febuxostat usage can range from 3 days to around 3 months. Neutropenia may develop during this timeframe. Administering symptomatic treatment can result in a gradual recovery within 1–2 weeks. Although there is limited information available regarding febuxostat‐induced agranulocytosis, it is crucial for healthcare professionals to remain alert and knowledgeable about this potential side effect. This awareness is vital for educational purposes and to further our comprehension of the condition. Additionally, the precise cause of this blood‐related toxicity remains unidentified, underscoring the necessity for ongoing monitoring to mitigate risks and improve medication safety protocols.

## Author Contributions

**Qiuping Liu:** writing – original draft, writing – review and editing. **Bing Zhong:** writing – review and editing.

## Consent

Written informed consent was obtained from the parents to publish this report in accordance with the journal's patient consent policy.

## Conflicts of Interest

The authors declare no conflicts of interest.

## Data Availability

All data and materials used in this study are available for sharing with the researchers. Please get in touch with the corresponding author.
